# A review on prospects, challenges, stakeholders and strategies for the implementation of school feeding programme in developing countries towards achieving Sustainable Development Goal 4

**DOI:** 10.4102/phcfm.v18i1.5348

**Published:** 2026-07-20

**Authors:** Esther Adekunle, Ferdinand Ottoh, Goddy Osimen, Adedotun Akinola

**Affiliations:** 1 Department of Political Science and International Relations, Covenant University, Ota, Nigeria; 2 Department of Political Science, University of Lagos, Yaba, Nigeria; 3 Department of Architecture, Covenant University, Ota, Nigeria

**Keywords:** quality education, school feeding programme, academic performance, student enrolments, SDG 4, sustainable development goals

## Abstract

School feeding have become increasingly important because of their contribution to achieving Sustainable Development Goal 4 (SDG 4). This study reviews literature on prospects, challenges, stakeholders and strategies for implementation of the SFP in developing countries to attain SDG 4. School feeding in developing countries improved learning and attendance, as well as nutrition. Major implementation challenges involved logistics, costs and political instability. The SFP could be implemented successfully if there were integrity of policy, community engagement and sustainable resources. This study adds to the literature by highlighting prospects, challenges, stakeholders and strategies confronting the implementation of school feeding in developing countries. Furthermore, the study will provide valuable information for researchers and policymakers in realising SDG 4.

## Introduction

Quality education provision remains a central concern in most developing countries, where school enrolment and academic achievement continue to be undermined by poverty, hunger and ill health. School feeding programmes (SFPs) have emerged as an extremely successful intervention to mitigate such concerns through meal delivery to school-age children, thus enhancing their nutritional status and ability to learn.^1^ In addition to their intrinsic value, SFPs have become increasingly importance because of their contributions to achieving Sustainable Development Goal 4 (SDG 4), which aims to ensure inclusive and equitable quality education and promote lifelong learning opportunities for all.^2^

In fact, SFPs supply the nutrients to combat malnutrition, mainly in the vulnerable groups. For instance, a healthy-eating child is better prepared for learning. Again, the habit of regular mealtimes has several benefits on general health in children, such as a reduced number of days off for sickness and higher attainment. The reason is that having a meal at school encourages sending children to this facility. It enhances enrolment and attendance rates.^3^ In addition, through the inclusion of the provision of at least one meal per day to all students, SFPs guarantee equity for low-income learners. Furthermore, these initiatives often link with the local communities, ensuring community building and education. An integrated approach to achieving sustainable development and quality education is exemplified by school feeding programmes (SFPs), which offer benefits that extend far beyond provisions of meals.^4^

School feeding programmes create positive feedback that benefits people, societies and economies by reducing hunger, improving health and increasing education. For the programmes to be successful and contribute to achieving sustainable development goals, there must be proper implementation with strong policy frameworks and community engagement.^4,5^ School feeding programmes are one of the effective study improvement strategies in developing nations to improve children’s health and education.^5^ Although increased attendance, improved academic results and reduced dropout are probable benefits that would justify the investment, issues such as money, logistics and political unrest still remain evident.^4^

Proper coordination of the many stakeholders involved, such as governments, international organisations, local communities and the private sector, significantly contributes to the effective functioning of the programme. School feeding programmes can, therefore, be of utmost importance in ensuring quality education and long-term growth for developing countries through overcoming challenges, establishing a partnership, and improving sustainability. With issues of poverty, hunger, access and inequity, this paper is of current importance as SFP is an intervention and strategy towards improved physical and mental health, impacting the family and communities as well as preventive primary health care. Therefore, this article analyses the prospects, challenges faced by stakeholders and strategies which are associated with SFPs in developing countries. This is to evaluate SFPs efficiency and sustainability toward the realisation of SDG 4 and offer suggestions on how to increase their effectiveness.

## Research methods and design

A narrative literature review methodology fits well in this research because this research aims to interpret, discuss and construct a theoretical insight from literature, rather than a comprehensive analysis of literature. An identical approach has been made within various high-profile studies.^6,7,8,9^ The literature search of various data bases (Research Gate, Google Scholar and Scopus) covered papers from the period 2016 to 2026 based on relevance to today’s school feeding policy issues in the school environment in low- and middle-income countries. It was limited to reviews in the English language only.

## Results and discussions

### Prospects of school feeding programmes in developing countries

School feeding programmes in developing nations foster social, economic and educational development. Besides providing food, the SFP also supports children, families and communities. One of the biggest benefits is enhanced nutrition for children. For example, malnutrition leads to stunted growth or iron, vitamin A and iodine deficiencies. In addition, wholesome meals encourage growth, mental development and prevent diseases like anaemia or rickets. Consequently, healthier children learn more, do not skip school, and engage more in class.^10,11^ These programmes further increase school enrolment, particularly in food-insecure communities. Hence, school meals encourage the sending of children to school by parents, with a special promotion of girls to close the gender gap. Succinctly put, continuous meals decrease the rate of absence and improve education.^12,13^

School feeding stimulates the local economies because it generates demand for food locally. For example, schools buy from farmers in the vicinity, thereby encouraging rural livelihood and food security. The programmes also create employment opportunities for farmers, cooks and transporters, thus increasing economic resilience.^12,14^ Apart from health, nutrition through school meals helps in the emotional well-being of a child. In addition, equal access to meals ensures belonging, thereby reducing stigma and enhancing mental well-being.^15^ Furthermore, nutrition education encourages healthy lifetime eating patterns, developing habits that deter obesity.^16^ Investment in school feeding halts the vicious cycle of poverty. Well-fed children are more likely to succeed academically, develop into productive and responsible citizens, contribute to economic growth, and secure a better future for themselves and communities. All this is made possible by political will coupled with funding sustainability, which unlocks the key.

### Challenges in implementing school feeding programmes in developing countries

School feeding programmes improve nutrition, education and child development in developing countries but are faced with numerous challenges that threaten their efficacy, sustainability and coverage. Financing is one of the major concerns. Budget deficits by governments and unpredictability of donor contributions make the guarantee of its long-term support impossible.^17^ Thus, the main problems with SFPs involve recurring costs to buy food, store it, and distribute it in an administrative manner, which becomes crippling for non-developed nations of the world.^18,19^ Logistical problems associated with poor rural road networks and conditions of storage will increase the process’s complexity and make it more prone to retardation, losses, and ineffectiveness.^17,20^ Corruption and political uncertainty pose risks to SFP because of misallocating funds and diversion of supplies, including interruption of service.^21,22^

Cultural and dietary considerations further complicate the process of implementing SFPs in developing countries. For instance, regional food preferences, religious restrictions and food taboos must be considered to enhance programme acceptability. Poor availability of food that is acceptable to the culture decreases participation.^23,24^ Food safety and hygiene also create a challenge because poor handling, poor storage and poor preparation can lead to food-borne illness.^25^ There is inadequately trained staff because most of the schools do not have capacity in terms of food handling, sanitation and programme administration, which leads to inefficiency and health-related problems.^19,26^ Also, parent and local leader involvement remains critical to the success of the programme, yet few programmes achieve high levels of participation among parents and local leaders, resulting in a lack of effectiveness and sustainability.^27^ School feeding programmes also remain unsustainable in the long term. This probably serves to explain their vulnerability to donor adjustments in priorities and changes in economic fortunes stemming from dependence on foreign aid. In effect, where funding is not guaranteed, with accompanying strong government backing, sustainability is not assured. A weak monitoring mechanism has been another wellspring of programme failure. Poor food quality, nutritional and attendance rates are compromised by inadequate collection of data on them, leading to impaired problem identification and attendant interventions.

For such barriers to be overcome, an integrated system comprising strong political will, adequate and ensured funding, improved infrastructure, capacity building, community involvement and finally effective monitoring and assessment is required. It is only when these barriers have been overcome that SFPs will realise full potential and thus be capable of providing the long-term health and educational benefits for children in developing countries.

### Key stakeholders in school feeding programmes

With SFPs, many stakeholders at different levels are involved in their proper planning, implementation and sustainability. For example, government agencies take a central role in policy decision-making, financing and coordination. In addition, national governments develop nutritional guidelines, budget allocations and provide support through legislation, while local governments carry out food distribution and community engagement.^12^ In developing countries, international donors and agencies such as World Food Programme (WFP), United Nations Children’s Fund (UNICEF) and other agencies such as United States Agency for International Development (USAID) and Department for International Development (DFID) offer important financing and technical support.^28,29^

Schools handle day-to-day affairs, safe food movement, and distribution. For example, Meal administration is conducted by kitchen staff, teachers and administrators while students offer information on meal acceptance.^30,31^ Furthermore, farmers and local food sources contribute fresh produce, improving food security and local food systems.^23^ Also, parents and communities support SFPs by encouraging school attendance, meal inclusion in education systems and food quality.^32^ In addition to that, community leaders are involved in raising awareness and building acceptance, with Parent-Teacher Associations supporting advocacy and fundraising.^32^

The private sector, through food firms and logistics companies, contributes through food provision contracts, Corporate Social Responsibility (CSR) investment and transport solutions, particularly where they have operations in rural areas.^33^ Non-governmental organisations (NGOs) and civil society organisations also add value to programme effectiveness by providing funding, influencing policy, training, lobbying and advocating that SFPs target longer-term health and educational goals.^1,33^

Stakeholder collaboration is indispensable in making SFPs a success, and calls for improved nutrition of children, education and school enrolment. Challenges could be overcome, and programme effectiveness improved through regular communication and good coordination.

### Strategies for the effective implementation of school feeding programmes

For successful SFPs in developing countries, strategic planning, implementation and sustainability are necessary. There must be an enabling policy environment. The government should harmonise SFPs with national policies on education, health and social welfare to ensure long-term budgeting through the national budget and the allocation of resources.^12^

Community participation improves efficiency. For example, planning and supervision should involve parents, teachers and community leaders. Partnerships with farmers, local enterprises and NGOs promote sustainability through resource and expertise sharing.^15,28^ Proper financial sustainability is critical. Governments need to secure frequent funding, diversify the sources like business and foreign donors and source locally to reduce costs and enhance food security.^34^

There is a need for a safe supply chain. This is because effective logistics networks, proper storage and food safety training for school staff ensure timely, regular delivery of meals.^32^ Furthermore, building capacity in the stakeholders ensures long-term programme success. Food safety, project management and monitoring training of teachers, nutritionists and government officials ensures quality in programmes. Parent education facilitates higher acceptance within the community.^26,35^

School lunches need to be culturally acceptable and nutritionally sound. Menu planning needs to be advised by nutrition specialists to provide for children’s nutritional requirements without promoting unhealthy, highly processed foods.^34^ With regards to monitoring and evaluation, monitor programme effects. Continued measurement of student health, attendance and quality of meals ensures accountability and programme improvement.

School feeding programmes have the potential to promote social inclusion, allowing equal access to food for all school children irrespective of gender or disability, and also improving enrolment in schools, which may include girls. The application of technology in managing finances, monitoring food and communication enhances efficiency and transparency. Government, community, NGOs and private enterprise collaboration secures sustainability for SFPs to make continued improvements in nutrition, education and social development.^36,37^

## Conclusion

The article highlights opportunities, challenges, stakeholders and strategies related to SFPs in the developing world. The several benefits that have accrued from SFPs relate to improvements in nutrition, school attendance, academic performance and stimulation of local economies and contributions toward long-term growth. School feeding programmes have thus become necessary in improving education and health. Funding deficiencies, logistical inefficiencies and cultural variations are among the challenges that must be surmounted if the full potential is to be reaped. In order to be successful, it requires collaboration among governments, international organisations, local farmers, school personnel and communities. Coordination should be good. For SFPs to be sustainable, there must be sound policies in place, supported by efficient logistics, diversification of funding, community involvement, capacity building and continued monitoring. This helps in ensuring the realisation of benefits in the long run, such as in the health, education and social development of the children. To the same effect, there needs to be a concerted effort by governments, NGOs, communities and the private sector for SFPs to become effective and sustainable.
